# Mating frequency mediates personality expression in facultatively polyandrous mites

**DOI:** 10.1186/s12915-025-02474-7

**Published:** 2025-11-27

**Authors:** Peter Schausberger, Shogo Usugi, Chenhao Wang, Norihide Hinomoto

**Affiliations:** 1 Laboratory of Ecological Information, Graduate School of Agriculture, Kyoto University, Sakyo-Ku, Kyoto, 606-8502 Japan; 2 Department of Behavioral and Cognitive Biology, University of Vienna, Djerassiplatz 1, Vienna, 1030 Austria

**Keywords:** Activity, Animal personality, Asset protection, Mating frequency, Mites, Monandry, Polyandry, Repeatability, Residual reproductive value, Sociability

## Abstract

**Background:**

Animal personalities are characterized by within-individual consistency linked to among-individual variability. Personality expression is often dependent on major life history events such as mating and the onset of reproduction. Here, we hypothesized that in facultatively polyandrous animals, multiple mating increases the females’ assets (i.e., residual reproductive value — RRV), due to direct and/or indirect benefits. Based on the predictions of the asset protection principle, higher RRV should promote behaviors that reduce the risk of fitness loss and hence mediate behavioral repeatability displayed in groups.

**Methods:**

We tested our hypothesis in group-living predatory mites, *Phytoseiulus persimilis*. Predatory mite females were presented with one or two mates, and their postmating repeatability in activity and sociability was evaluated in groups composed of females of the same and mixed mating types.

**Results:**

Mating frequency had little effect on activity patterns but pronounced effects on sociability traits. Polyandrous females were on average more sociable as well as more repeatable in sociability than monandrous females. These behavioral shifts reflect strategies to mitigate inter-individual conflicts within groups to enhance asset protection.

**Conclusions:**

Our study suggests that the mating frequency can critically influence female personality expression after mating and highlights the importance of considering mate-related variables in animal personality research.

**Supplementary Information:**

The online version contains supplementary material available at 10.1186/s12915-025-02474-7.

## Background

Animal personalities are characterized by consistent within-individual behaviors that consistently differ among individuals across time and contexts [1, 2]. Animal personalities are not bound to certain taxonomic groups but are observed across animals, from anemones, arachnids, and insects to birds, fish, and mammals [3, 4]. Typical pathways mediating personality expression are genetic determination, transgenerational information transfer, and personal experiences. In many animals, personalities are not fixed throughout life but change over the course of ontogeny and/or are contingent upon major life history transitions or events [5], such as when being released from parental care, when becoming adult, when mating and starting to reproduce, or when ceasing reproduction [6–8].

The influence of personality expressed before or during mating on sexual selection and mating is relatively well researched [9, 10], but the influence of mating-associated events on female personality expression after mating remains elusive. Mating-related circumstances such as mate search and choice, the mating event itself, mating frequency, and the types of mates are some of the most critical events for any sexually reproducing animal, being decisive determinants of reproductive success and fitness. However, the proximate and ultimate aspects of mating-mediated adjustments in personality expression are poorly understood. This is also true for the role of the quality and/or quantity of male mates on female personality expression, yet the few studies that are available suggest a potentially larger influence than anticipated. For example, Monestier and Bell (2020) [11] showed that both mating and the mere presence of a courting male critically change the mean expression and repeatability of boldness and sociability of stickleback females, *Gasterosteus aculeatus*, as compared to no mating experience. Schausberger and Nguyen (2024) [12] observed that the early-life social experience of male mates critically influences the repeatability of activity and sociability of predatory mite females after mating. In polyandrous animals, initial mate choice and first mating experience are often decisive for subsequent choice and re-mating willingness [13]. Females typically become more selective with mating experience, which in turn should affect their personality expression. The influence of mating frequency on female personality expression is unknown for any animal.

Powerful ultimate explanations for mating-related adjustments of female personalities can be derived from the asset protection principle [14]. The asset protection principle postulates that individuals with many or valuable assets, i.e., high residual reproductive value (RRV), should avoid risks to allow harvesting these assets, while individuals with few or cheap assets, i.e., low RRV, should take more risks because they have less to lose [14]. Accordingly, mating-related experiences should influence mean behavioral trait expression as well as personality traits such as boldness (risk-taking behavior) [15]. While the asset protection principle has been originally developed [14] and evaluated [16, 17] for predation risk-taking, it is applicable to any behavior that is fitness relevant and bears some kind of risk of fitness loss/decrease [15], including the canonical personality traits aggressiveness and sociability, especially in group-living animals.

Here, we tested the hypotheses that in animals with facultative polyandry, which is a widespread mating system [13, 18–20], multiple mating increases the asset quality (RRV), compared to single mating, and should thus lead to more risk-averse behaviors (in a broad sense: risk of fitness loss). Multiple mating can increase the asset quantity through direct (material) and asset quality through indirect (genetic) benefits [13, 18, 21]. Multiple mating should diversify the females’ behaviors, because each female experiences a different set of two mates with presumably different qualities, compared to single mating, which could in turn result in lower within-individual variability relative to among-individual variability, together increasing behavioral repeatability. More or higher quality assets (higher RRV) should be linked to increased behavioral repeatability if the asset quantity or quality cannot be increased anymore (no search or choice of additional mates needed; no mate competition; diversity among females is promoted because different sets of two mates provide more complex information to females and allow much more variability than single mates). Increased assets should result in more consistent within-individual behaviors, relative to among-individual variability, if within-individual consistency coupled to among-individual variability and asset value are linked by positive feedback loops [22–24].

We tested our hypotheses in plant-inhabiting predatory mites *Phytoseiulus persimilis*. These mites are adapted to live in groups, which is brought about by the patchy distribution of their prey (spider mites) and mutual attraction [25–28]. Compromised sociability and social competence interfere with group cohesion, increase inter-individual conflicts, and thus pose a risk to fitness [12, 27, 28]. *P. persimilis* females are facultatively polyandrous, i.e., they mate once or twice and store the spermatophores for life [29]. Monandrous and polyandrous females produce similar numbers of eggs, but the second mate provides indirect benefits, due to allowing sperm competition and offspring sired by two males [29]. In cases where first males fail to transfer sperm, second males provide direct benefits through fertilization assurance [29]. Higher mating frequencies than two are rarely observed in *P. persimilis* [29], because excess mating has only costs [30] but no benefits (excess males do not sire any offspring in *P. persimilis* [31]). Having a second mate enhances the asset quality for *P. persimilis* females because their offspring become genotypically and phenotypically more diverse [29], which is commonly considered a major indirect benefit of multiple mating [18–20]. Such offspring thus constitute, as a whole, a higher RRV for their mothers. In *P. persimilis*, higher asset quality of heterogeneous offspring is linked to group living and the associated risks of inbreeding and niche overlap. Offspring tend to stay in the natal patch until mating [32]. However, mating between full siblings leads to inbreeding depression [33–35], and, under certain circumstances, full siblings become the fiercest competitors [36]. Higher genotypic and phenotypic diversity at the local scale mitigates the costs of inbreeding [29, 33–35], allows more favorable mate choice [37], enhances individual niche segregation [12], and is advantageous in unpredictable environments, as a bet-hedging strategy [29].

## Results

**Fig. 1:**
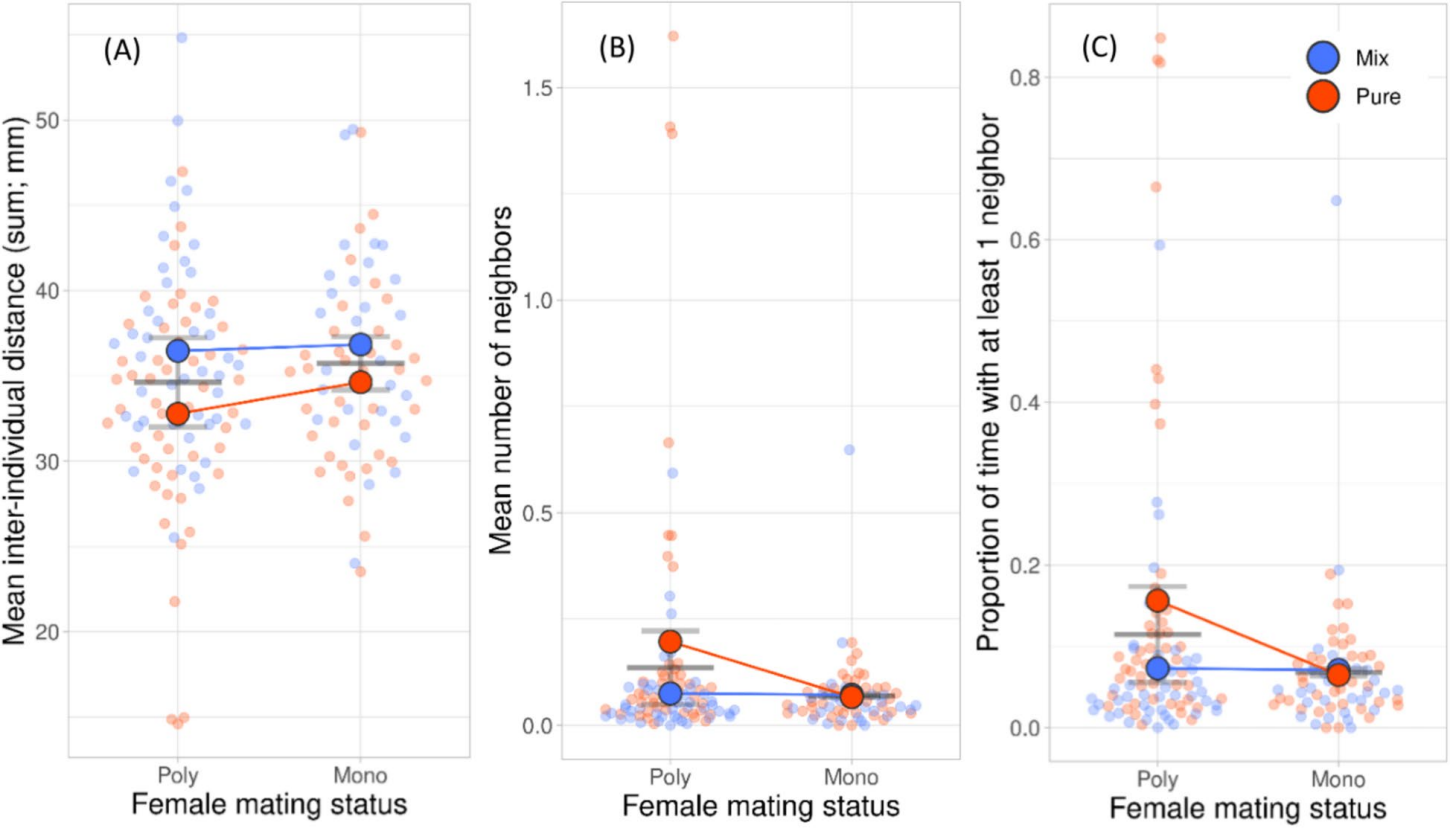
Mean (± SE) inter-individual distance (**A**), mean number of neighbors (**B**, within a radius of 2 mm of the target), and mean proportion of time with at least one neighbor (**C**) of *P.* *persimilis* females, which had mated once (mono, *N* = 28) or twice (poly, *N* = 44), sequentially observed in groups of four females with the same (pure) and different (mix) mating frequency. Pale dots are the individual data (Additional File 1: Table S1)

**Fig. 2:**
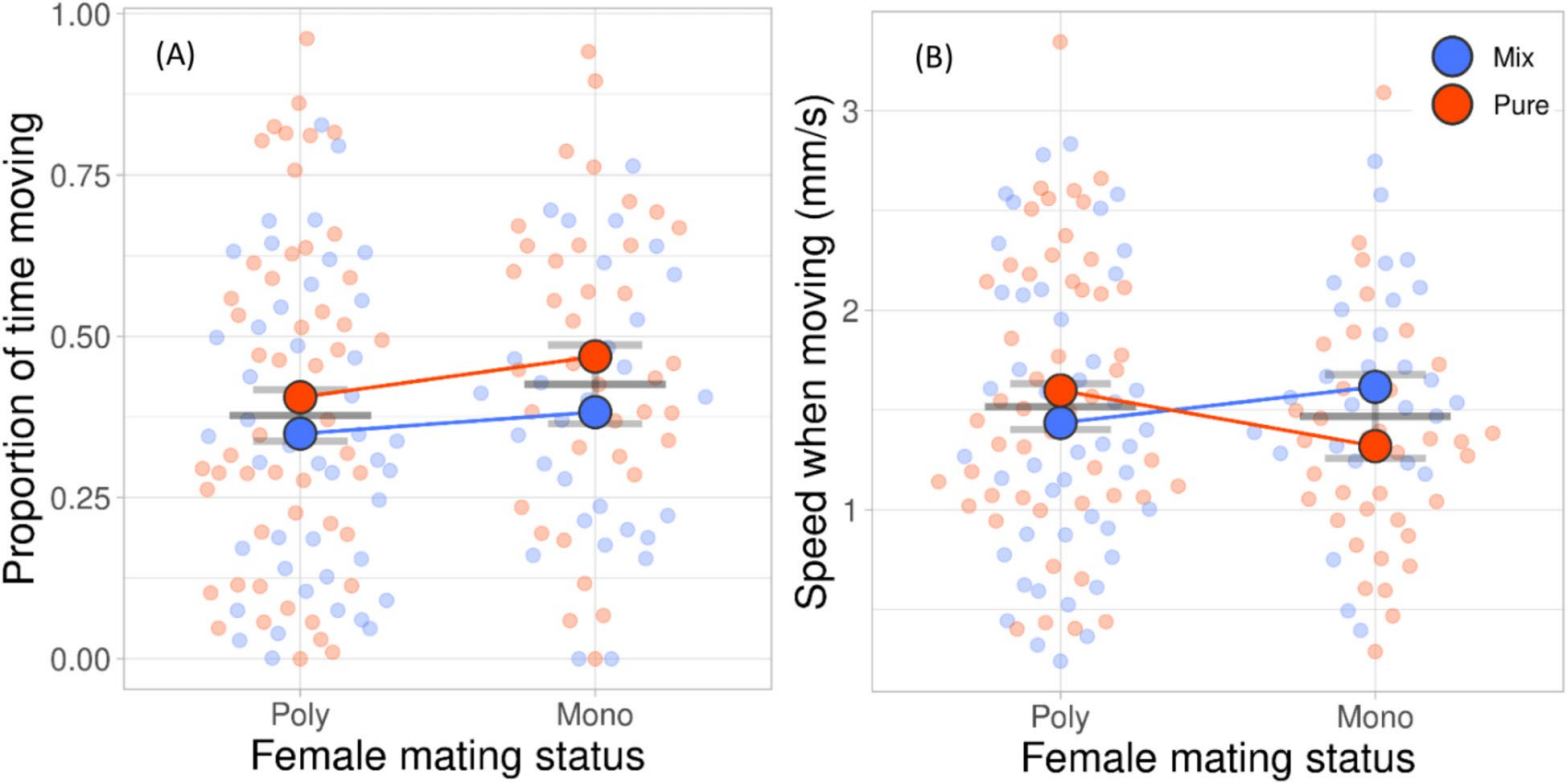
Mean (± SE) proportion of time moving (**A**) and mean running speed when moving (**B**) of *P.* *persimilis* females, which had mated once (mono; *N* = 28 in **A**, 26 in **B**) or twice (poly; *N* = 44 in **A**, 42 in **B**), sequentially observed in groups of four females with the same (pure) and different (mix) mating frequency. Pale dots are the individual data (Additional File 1: Table S1)

Type of group assay (pure or mixed group) had a significant effect on the mean inter-individual distance, number of immediate neighbors (within a radius of 2 mm of the center of the target), proportion of time with at least one neighbor, and proportion of time moving of *P. persimilis* females (Table 1, Figs. 1A, B, and C and 2A). Females were closer together (Fig. 1A), had more neighbors (Fig. 1B), spent more time with at least one neighbor (Fig. 1C), and moved more (Fig. 2A) in pure than mixed groups. However, having more neighbors and spending more time with at least one neighbor in pure than mixed groups was only the case in polyandrous but not monandrous females (Fig. 1B, C). The number of eggs produced on the leaf discs did not differ between monandrous (11.79 ± 0.80 SE) and polyandrous (12.50 ± 0.66 SE) females (GLM: Wald chi-square = 0.460, *P* = 0.498). Polyandrous females ran faster in pure than mixed groups, whereas the opposite was true for monandrous females (Fig. 2C). Polyandrous but not monandrous females were repeatable in inter-individual distance, number of immediate neighbors, and time spent with at least one neighbor (Table 2). Neither type of female was repeatable in the proportion of time moving and running speed (Table 2). Scrutiny of the among- and within-individual variances of the sociability traits revealed that the higher repeatability of polyandrous than monandrous females was due to the following: (i) For inter-individual distance, a combination of higher among-individual variability and higher within-individual consistency and smaller within-variability than among-variability in polyandrous but not monandrous females, and (ii) for the number of neighbors and the proportion of time with at least one neighbor, smaller within-variability than among-variability in polyandrous females, whereas the within-variability and among-variability were similar in monandrous females. The variances in sociability traits had different levels in polyandrous and monandrous females, with higher levels in polyandrous females. Both variances were extremely small and similar in monandrous females, suggesting that all females behaved very similar; polyandrous females diversified more among each other, but behaved, in relative comparison of among- and within-variability, more consistent within individuals.

**Table 1:** Results of generalized estimating equations (GEE) of the effects of the mating frequency (once or twice) of *P. persimilis* females and the type of group assay (pure or mixed). Each female was sequentially observed in a group of four females with the same (pure, all either monandrous or polyandrous) and different (mixed, two monandrous and two polyandrous) mating status

Dependent variable	Independent variables	*N*	Wald	df	*p*-value
			**Chi-square**		
Inter-individual distance	(Intercept)	161	5222.209	1	<.001
	Mating frequency		1.423	1	0.233
	**Type of assay**		**10.663**	**1**	**0.001**
	Mating frequency × type of test		0.698	1	0.404
Number of neighbors	(Intercept)	161	41.577	1	<.001
	**Mating frequency**		**4.454**	**1**	**0.035**
	**Type of assay**		**5.637**	**1**	**0.018**
	**Mating frequency × type of assay**		**6.805**	**1**	**0.009**
Time with ≥ 1 neighbor	(Intercept)	161	64.132	1	<.001
	**Mating frequency**		**4.152**	**1**	**0.042**
	**Type of assay**		**4.799**	**1**	**0.028**
	**Mating frequency × type of assay**		**6.584**	**1**	**0.010**
Time moving	(Intercept)	161	463.104	1	<.001
	Mating frequency		1.657	1	0.198
	**Type of assay**		**4.062**	**1**	**0.044**
	Mating frequency × type of assay		0.200	1	0.655
Running speed	(Intercept)	157	850.683	1	<.001
	Mating frequency		0.208	1	0.648
	Type of assay		0.476	1	0.490
	**Mating frequency × type of assay**		**5.362**	**1**	**0.021**

Significant effects (*P* < 0.05) are highlighted in bold

**Table 2:** Intraclass correlation coefficients (ICC, average measure; repeatability) of behavioral traits of *P. persimilis* females as affected by the mating frequency (mono- or polyandrous). Each female was sequentially observed in a group of four females with the same (pure, all either monandrous or polyandrous) and mixed (two monandrous and two polyandrous) mating status

Personality trait	Mating frequency	*N*	ICC	CI (95%)	*p*-value
Inter-individual distance	Mono	28	− 0.272	− 1.750, 0.411	0.732
	**Poly**	**44**	**0.497**	**0.078, 0.726**	**0.013**
Number of neighbors	Mono	28	− 0.098	− 1.374, 0.492	0.595
	**Poly**	**44**	**0.493**	**0.070, 0.723**	**0.014**
Time with ≥ 1 neighbor	Mono	28	− 0.080	− 1.334, 0.500	0.579
	**Poly**	**44**	**0.578**	**0.227, 0.770**	**0.003**
Time moving	Mono	28	− 0.039	− 1.245, 0.519	0.539
	Poly	44	0.233	− 0.407, 0.581	0.195
Running speed	Mono	26	0.379	− 0.384, 0.722	0.120
	Poly	42	− 0.306	− 1.430, 0.298	0.802

Significant effects (*P* < 0.05) are highlighted in bold

## Discussion

Our study documents that the mating frequency mediates personality expression in facultatively polyandrous predatory mites *P. persimilis*. Polyandrous but not monandrous females were repeatable in sociability traits such as inter-individual distance, number of immediate neighbors, and proportion of time spent with at least one neighbor. High repeatability in sociability traits of polyandrous females was primarily due to lower within-individual variances than among-individual variances. In monandrous females, and depending on the trait, within- and among-variances were similar, or among-variance was higher than within-individual variances, compromising repeatability. Polyandrous females were not only more repeatable but, on average, also more sociable than monandrous females. The type of group composition (purely monandrous/polyandrous or mixed) influenced the mean behavioral trait expressions in activity and sociability traits but more so in polyandrous than monandrous females.

Mating frequency was a previously unknown factor mediating the behavioral repeatability of females following mating. Other mating-related factors were documented for sticklebacks [11] and the very same animals of this study, predatory mites *P. persimilis* [12]. Sticklebacks became, on average, less sociable and less risk-taking after mating or mere courtship by males; also, the repeatability of sociability and boldness before and after the mating opportunity was lower than in the unmated control group [11]. Female sticklebacks compete for placing their eggs inside the nests built by males, which may explain why they became less sociable and more competitive after mating. Our study animals, the group-living *P. persimilis*, became both on average more sociable and more repeatable in sociability following the second mating. Such behavioral shifts should enhance fitness in group-living species. Reproducing *P. persimilis* females benefit from joint foraging and spider mite patch exploitation, with these benefits being higher than the costs of competition. Group living is the norm in *P. persimilis*; only during dispersal and low prey availability, or when the previously ample prey supply deteriorates, do the groups dissolve.

From a proximate perspective, the study by Monestier and Bell (2020) [11] suggests that in sticklebacks, the mere social experience of being courted by a male can alter the repeatability of female behavior, with the social and sexual (mating) experiences exerting the same effects. Our experimental procedure presented the females one (for the monandrous treatment) or two males (for the polyandrous treatment), with the duration of each male presentation allowing only for one complete mating, typically lasting 2 to 3 h [29, 37, 38]. All females mated once, as indicated by egg production after the first mating. All females of the polyandry treatment experienced the presence of a second mating partner, and we assume that most of them, if not all, did mate twice. Typically, around 90% of *P. persimilis* females do mate upon presentation of a second mate, with around 70% of these producing offspring with mixed paternities [29]. We consider it highly unlikely that the mere presence of a male or courtship alone would have induced similar behavioral changes as the mating experience did. The reason is that, unlike stickleback males who court females elaborately for an extended period of time and fertilize the eggs externally, male *P. persimilis* transfer sperm directly, and the courtship period is extremely short relative to the copulation duration [39].

Ultimate explanations for why multiple mating produced more repeatable females (more variable among individuals but, in relative comparison, more consistent within individuals) can be inferred from mate choice and competition and asset (RRV) protection [14]. Mate choice and competition are over for polyandrous females, whereas monandrous females still compete for additional mates [35, 37]. This also explains why polyandrous females were on average more sociable in groups consisting of just polyandrous females but not in mixed groups, consisting of both monandrous and polyandrous females. Polyandrous females are not engaging anymore in mate choice or competition with other females and thus have lower needs to flexibly adjust their behavior to the behavior of others. Due to their higher RRV, polyandrous females should be more risk averse than monandrous females and behave in a way that limits the risk of fitness loss. They should be more protective of their offspring than monandrous females and optimize their realized niches, including the interactions with conspecifics. By predictably signaling their state to other cohabiting females, they avoid the costs of mate competition; by signaling their state to cohabiting males, they avoid the costs of mating attempts that would not result in any direct or indirect benefits [29–31]. Higher variability among polyandrous than monandrous females was likely brought about by the influence of different sets of two mates, providing highly variable information to females. Increased among-individual variability, reflecting individualized female behaviors, should reduce intragroup conflicts. Similar to the large difference in repeatability of monandrous and polyandrous females in this study, Schausberger and Nguyen (2024) [12] observed strongly differing repeatabilities in females mediated by the early-life experiences of their mating partners. Male mates that had been socially isolated early in life strongly increased the repeatability in activity of females that had been grouped early in life [12].

## Conclusions

Our study underscores the critical importance of the females’ mates on female personality expression after mating. Together with Monestier and Bell (2020) [11] and Schausberger and Nguyen (2024) [12], our study demonstrates the sensitivity of adult female personality formation to mate-related circumstances, such as the quantity and phenotypes of mates, mediating mean trait expression as well as behavioral repeatability after mating. Our study suggests that mating frequency and other mating-related aspects deserve more attention in animal personality studies for being potential drivers of behavioral repeatability in sexually reproducing animals, with significant ecological and evolutionary implications.

## Methods

### Predatory mite origin and rearing

Predatory mites, *P. persimilis*, used in experiments were derived from a laboratory-reared population that had been founded with specimens collected on eggplant, *Solanum melongena*, in Sicily [12]. In the laboratory, the predatory mites were reared in heaps of detached leaves of common bean plants, *Phaseolus vulgaris*, infested with two-spotted spider mites *Tetranychus urticae*, inside small plastic boxes (11.7 × 16.5 × 5.6 cm). The small plastic boxes were fixed inside large plastic boxes (23.9 × 17.6 × 9.1 cm), containing a shallow layer of soapy water, and closed on top by a lid with a mesh-covered rectangular opening (8 × 10 cm) for ventilation. The rearing boxes were stored in an air-conditioned room at 25 ± 1 °C, 60 to 80% relative humidity (RH), and 16:8 h light:dark (L:D). The spider mite population was founded by specimens collected from chrysanthemum plants, *Chrysanthemum morifolium*, in Nara, Japan, and reared on whole common bean plants at room temperature under daylight fluorescent lamps (16:8 h L:D). Spider mite-infested bean leaves were clipped off the plants and added to the small boxes used for rearing the predatory mites twice per week.

### Pre-experimental treatments

To generate predatory mite females used in experiments, gravid females (recognizable by their expanded body) were randomly withdrawn from the rearing and placed in groups of 50 on detached spider mite-infested bean leaf arenas. Each arena consisted of a primary leaf (~ 35 cm^2^) placed upside down on moist filter paper on top of a circular foam pad resting inside a Petri dish (9 cm Ø) half-filled with water. Moist tissue paper was wrapped around the edges of the leaf arena. Predatory mite eggs < 24 h old were collected and transferred in groups of 50 to fresh large leaf arenas, infested with spider mites, for development; leaf arenas were monitored once per day until the predatory mites had reached the deutonymphal stage. Female deutonymphs were removed and singly placed on circular bean leaf discs (1.2 to 1.5 cm Ø), which harbored mixed spider mite stages, resting upside down on water agar columns (1.2 to 1.5 cm Ø, 1.1 cm high) inside closed acrylic cages half-filled with water. Each acrylic cage consisted of a closed Petri dish (5.0 cm Ø, height 1.5 cm) with a mesh-covered (mesh size 0.05 mm) ventilation opening (1.3 cm Ø) in the lid (SPL Life Sciences Co. Ltd., South Korea). As soon as the females were adult, one adult male, randomly taken from another large leaf arena than the female came from, was added to the leaf disc and left there for ~ 3 h. Mating typically commences within 30 to 60 min after pairs are together on a leaf and lasts on average around 2 to 3 h [29, 37, 38]. After ~ 3 h, the male was removed, while the female was left on the disc. After another 2 days, half of the females received a second male for mating for 5 to 6 h [31] (to represent polyandrous females in the experiment), while the other half of the females was left without a second male (to represent monandrous females in the experiment). Sample sizes at the start of the behavioral assays were 37 monandrous females and 50 polyandrous females. Monandrous and polyandrous females had the same age and were always tested in parallel in the behavioral assays. Eggs were counted and removed from the discs in 1- to 2-day intervals. All experimental units were stored in an air-conditioned room at 25 ± 1 °C, 60 to 80% RH, and 16:8 h L:D.

### Behavioral assays

Each monandrous and polyandrous female was subjected to two group assays, dubbed “pure” and “mixed,” using rectangular acrylic arenas (2.0 × 1.1 cm). Each arena was bordered by 1 to 2 mm high walls, built of water-soaked cotton pads, and preloaded with ~ 60 eggs of two-spotted spider mites, spread equally on the surface of the arena before conducting the assay. In pure group assays, four females with the same mating type were grouped together in each arena (either four monandrous or four polyandrous females). In mixed group assays, two monandrous and two polyandrous females were grouped together in each arena. Before assays, each predatory mite female was uniquely colored by a small watercolor dot on her dorsal shield, applied by a marten’s hair brush (size 0), to make her identifiable during the assays. To start a group assay, four differently colored females were transferred from their leaf discs to the arena and allowed to acclimatize for 5 min. After 5 min, the behavior of the females was videotaped for 4 min using either a USB digital microscope (Jiusion HD 2MP, Shenzhen Jiu Sheng Electronic Commerce, Shenzhen, China) or a color CMOS camera (WRAYCAM-NOA2000, Wraymer, Osaka, Japan) attached to a stereo microscope (Olympus SZ61TRC-C, Tokyo, Japan). After the group assays, the females were immediately returned to the leaf disc they came from. Mixed group assays took place 2 days after the pure group assays (i.e., females had a 2-day rest in between the pure and mixed group assays).

### Video-tracking and statistical analysis

Videotaped behaviors (15 frames/s in each video) were automatically analyzed using AnimalTA, version 3.2.1 [40]. AnimalTA allows tracking the movement and inter-individual interactions separately for each individual of a group. The trajectories of each individual target obtained by video-tracking were used for analysis of the proportion of time moving (moving threshold was 0.2 mm/s), running speed when moving, inter-individual distance, number of immediate neighbors (i.e., within a radius of 2 mm from the target, which is the touching distance when both interactants longitudinally stretch their first pair of legs [41]), and the proportion of time spent with at least one neighbor.

All statistical analyses were conducted using IBM SPSS Statistics v. 29.0.1 [42]. All raw data are in Additional file 1: Table S1. Separate generalized estimating equations (GEEs, linear), which account for the interdependency of repeated tests, were used to analyze the effects of female mating frequency (mono- or polyandrous) and type of group assay (pure or mixed) on the mean proportion of time spent moving, running speed, inter-individual distance, number of neighbors, and proportion of time with at least one neighbor. A generalized linear model (GLM, linear) was used to examine the effect of female mating frequency on the number of eggs produced on their leaf discs over 5 days. Personality expression by monandrous and polyandrous females, i.e., behavioral repeatability between group assays, in the proportion of time spent moving, running speed, inter-individual distance, number of neighbors, and proportion of time with at least one neighbor was evaluated by intraclass correlation coefficients (ICC; two-way random, consistency, average measure [43]). Within- and among-individual variances were checked to pinpoint the causes of different ICCs induced by female mating frequency (mono- or polyandrous). SuperPlotsOfData [44] was used to create Figs. 1 and 2.

## Supplementary Information

Additional File 1: Table S1. Raw data of the experiment.

## Data Availability

All data generated and analyzed during this study are included in the manuscript and its supplementary files (Additional file 1: Table S1).

## Abbreviations

RRV: Residual reproductive value
GLM: Generalized linear model
RH: Relative humidity
L: Light
D: Dark
GEE: Generalized estimating equations
ICC: Intraclass correlation coefficient

## References

[1] Sih A, Bell A, Johnson JC. Behavioral syndromes: an ecological and evolutionary overview. Trends Ecol Evol. 2024;19:372–278. 10.1016/j.tree.2004.04.009 16701288

[2] Réale D, Reader SM, Sol D, McDougall PT, Dingemanse NJ. Integrating animal temperament within ecology and evolution. Biol Rev. 2007;82:291–318. 17437562 10.1111/j.1469-185X.2007.00010.x

[3] Bell AM, Hankison SJ, Laskowski K. The repeatability of behavior: a meta-analysis. Anim Behav. 2009;77:771–83. 24707058 10.1016/j.anbehav.2008.12.022 PMC3972767

[4] Carere C, Maestripieri D. Animal personalities: behavior, physiology, and evolution. University of Chicago Press; 2013.

[5] Stamps J, Groothuis TGG. The development of animal personality: relevance, concepts and perspectives. Biol Rev. 2010;85:301–25. 19961473 10.1111/j.1469-185X.2009.00103.x

[6] Wilson ADM, Krause J. Personality and metamorphosis: is behavioral variation consistent across ontogenetic niche shifts? Behav Ecol. 2012;23:1316–23.

[7] Cabrera D, Nilsson JA, Griffen BD. The development of animal personality across ontogeny: a cross-species review. Anim Behav. 2021;173:137–44.

[8] Fortunato JA, Earley RA. Age-dependent genetic variation in aggression. Biol Lett. 2023;19:20220456. 36693426 10.1098/rsbl.2022.0456 PMC9873472

[9] Schuett W, Tregenza T, Dall SRX. Sexual selection and animal personality. Biol Rev. 2010;85:217–46. 19922534 10.1111/j.1469-185X.2009.00101.x

[10] Munson AA, Jones C, Schraft H, Sih A. You’re just my type: mate choice and behavioral types. Trends Ecol Evol. 2020;35:823–33. 32451175 10.1016/j.tree.2020.04.010

[11] Monestier C, Bell AM. Personality traits change after an opportunity to mate. Proc R Soc B. 2020;287:20192936. 32345156 10.1098/rspb.2019.2936 PMC7282925

[12] Schausberger P, Nguyen T-H. Early social isolation disrupts personality expression in group-living mites. J Anim Ecol. 2025;94:45–57. 39180272 10.1111/1365-2656.14169 PMC11730261

[13] Jennions MD, Petrie M. Variation in mate choice and mating preferences: a review of causes and consequences. Biol Rev. 1997;72:283–327. 9155244 10.1017/s0006323196005014

[14] Clark CW. Antipredator behavior and the asset-protection principle. Behav Ecol. 1994;5:159–70.

[15] Wolf M, van Doorn GS, Leimar O, Weissing FJ. Life-history trade-offs favour the evolution of animal personalities. Nature. 2007;447:581–4. 17538618 10.1038/nature05835

[16] Moschilla JA, Tomkins JL, Simmons LW. State-dependent changes in risk-taking behaviour as a result of age and residual reproductive value. Anim Behav. 2018;142:95–100.

[17] Dammhahn M. Are personality differences in a small iteroparous mammal maintained by a life-history trade-off? Proc R Soc B. 2012;279:2645–51. 10.1098/rspb.2012.0212 PMC3350711 22398164

[18] Arnqvist G, Nilsson T. The evolution of polyandry: multiple mating and female fitness in insects. Anim Behav. 2000;60:145–64. 10973716 10.1006/anbe.2000.1446

[19] Gowaty PA. Adaptively flexible polyandry. Anim Behav. 2013;86:877–84.

[20] Pizzari T, Wedell N. The polyandry revolution. Philos Trans R Soc B. 2013;368:20120041. 10.1098/rstb.2012.0041 PMC3576576 23339233

[21] Ridley M. Mating frequency and fecundity in insects. Biol Rev. 1988;63:509–49.

[22] Dall SRX, Houston AI, McNamara JM. The behavioural ecology of personality: consistent individual differences from an adaptive perspective. Ecol Lett. 2004;7:734–9.

[23] McElreath R, Luttbeg B, Fogarty SP, Brodin T, Sih A. Evolution of animal personalities. Nature. 2007;450:E5. 17994035 10.1038/nature06326

[24] Sih A, Mathot KJ, Moirón M, Montiglio P-O, Wolf M, Dingemanse NJ. Animal personality and state-behaviour feedbacks: a review and guide for empiricists. Trends Ecol Evol. 2015;30:50–60. 25498413 10.1016/j.tree.2014.11.004

[25] Sabelis MW. Development. In: Helle W, Sabelis MW, editors. Spider mites. Their biology, natural enemies and control, Vol 1B. Amsterdam: Elsevier; 1985. p. 43–53.

[26] Zhang Z-Q, Sanderson JP. Short distance location of spider mite colonies by three predatory mites (Acari, Tetranychidae, Phytoseiidae) - predator responses to prey-associated and predator-associated stimuli. Environ Entomol. 1992;21:801–7.

[27] Muleta MG, Schausberger P. Smells familiar: group-joining decisions of predatory mites are mediated by social familiarity. Anim Behav. 2013;86:507–12. 24027341 10.1016/j.anbehav.2013.05.040 PMC3763367

[28] Strodl MA, Schausberger P. Social familiarity relaxes the constraints of limited attention and enhances reproduction of group-living predatory mites. Oikos. 2013;122:1217–26. 24273345 10.1111/j.1600-0706.2012.20833.x PMC3837212

[29] Schausberger P, Patiño-Ruiz JD, Osakabe M, Murata Y, Sugimoto N, Uesugi R, et al. Ultimate drivers and proximate correlates of polyandry in predatory mites. PLoS One. 2016;11:e0154355. 27100395 10.1371/journal.pone.0154355 PMC4839743

[30] Daly M. The cost of mating. Am Nat. 1978;112:771–4.

[31] Walzer A, Schausberger P. Canalization of body size matters for lifetime reproductive success of male predatory mites (Acari: Phytoseiidae). Biol J Linn Soc. 2014;111:889–99. 10.1111/bij.12235 PMC4133644 25132689

[32] Sabelis MW, Dicke M. Long-range dispersal and searching behaviour. In: Helle W, Sabelis MW, editors. Spider mites. Their biology, natural enemies and control, Vol 1B. Amsterdam: Elsevier; 1985. p. 141–157.

[33] Tregenza T, Wedell N. Polyandrous females avoid costs of inbreeding. Nature. 2002;415:71–3. 11780118 10.1038/415071a

[34] Atalay D, Schausberger P. Balancing in- and out-breeding by the predatory mite *Phytoseiulus persimilis*. Exp Appl Acarol. 2018;74:159–69. 29460092 10.1007/s10493-018-0225-3 PMC5847215

[35] Schausberger P, Cekin D. Plastic female choice to optimally balance (k)in and out-breeding in a predatory mite. Sci Rep. 2020;10:7861. 32398794 10.1038/s41598-020-64793-9 PMC7217829

[36] Schausberger P. Ontogenetic isolation favors sibling cannibalism in mites. Anim Behav. 2004;67:1031–5.

[37] Enigl M, Schausberger P. Mate choice in the predaceous mite Phytoseiulus persimilis: evidence of self-referent phenotype matching? Entomol Exp Appl. 2004;112:21–8.

[38] Lv J, Zhang B, Jiang X, Wang E, Xuenong X. Quantitative impact of mating duration on reproduction and offspring sex ratio of Phytoseiulus persimilis (Acari: Phytoseiidae). J Integr Agric. 2019;18:884–92.

[39] Amano H, Chant DA. Mating behaviour and reproductive mechanisms of two species of predacious mites, *Phytoseiulus persimilis* Athias-Henriot and *Amblyseius andersoni* (Chant) (Acarina: Phytoseiidae). Acarologia. 1979;20:196–213.

[40] Chiara V, Kim S-Y. AnimalTA: a highly flexible and easy-to-use program for tracking and analysing animal movement in different environments. Methods Ecol Evol. 2023;14:1699–707.

[41] Schausberger P, Luh H-K, Croft BA. Larval size relative to larval feeding, cannibalism of larvae, egg or adult female size and larval–adult setal patterns among 13 phytoseiid mite species. Exp Appl Acarol. 1999;23:599–610.

[42] IBM Corporation. IBM SPSS statistics for windows, version 29.0.1.0. IBM Corporation; 2023.

[43] McGraw KO, Wong SP. Forming inferences about some intraclass correlation coefficients. Psychol Methods. 1996;1:30–46.

[44] Goedhart J. Superplotsofdata—a web app for the transparent display and quantitative comparison of continuous data from different conditions. Mol Biol Cell. 2021;32:470–4. 33476183 10.1091/mbc.E20-09-0583 PMC8101441

